# Personal protective measures of pregnant women against Zika virus infection

**DOI:** 10.11606/s1518-8787.2019053001146

**Published:** 2019-08-23

**Authors:** Vladimir Antonio Dantas Melo, José Rodrigo Santos Silva, Roseli La Corte

**Affiliations:** I Universidade Federal de Sergipe. Programa de Pós-Graduação em Biologia Parasitária. São Cristovão, SE, Brasil; II Universidade Federal de Sergipe. Departamento de Estatística e Ciências Atuariais. Centro de Ciências Exatas e Tecnologia. São Cristovão, SE, Brasil; III Universidade Federal de Sergipe. Departamento de Morfologia. Centro de Ciências Biológicas e da Saúde. São Cristovão, SE, Brasil

**Keywords:** Pregnant Women, Zika Virus Infection, prevention & control, Arbovirus Infections, prevention & control, Insect Repellents, Mosquito Nets, utilization, Socioeconomic Factors

## Abstract

**OBJECTIVE:**

To evaluate the adherence of pregnant women to personal protective measures against mosquito bites, recommended by the Ministry of Health, and to investigate the factors associated with the non-adoption of these measures.

**METHODS:**

We interviewed 177 pregnant women between November 2016 and February 2017 in the 10 basic health units of the municipality of Propriá, state of Sergipe, two located in the rural area and eight in the urban area, during prenatal appointments, to raise information about the use of preventive measures against the vector transmission of Zika virus. Data were analyzed using descriptive statistical methods, chi-square test or Fisher’s exact test, and the odds ratio was calculated. The independent variables were grouped by the analysis of principal components, and the dependents (the use of repellent, mosquito nets, garments, screens and insecticides) were analyzed using the logistic regression method.

**RESULTS:**

Among the measures recommended by the Ministry of Health, mosquito nets were the most used by pregnant women living in rural areas and with low education level, while the repellents were more used by women in the urban area and with higher education level. Women in a vulnerable socio-economic situation presented a risk 2.4 times higher for not using screens in their homes, 1.9 times higher for not changing clothes and 2.5 times higher for not using repellent than pregnant women in better economic conditions.

**CONCLUSIONS:**

The socioeconomic status of pregnant women, especially among the less privileged, influenced the use of protective measures against Zika virus, from the purchase of repellent, clothing, insecticides to other resources in the municipality of Propriá, SE.

## INTRODUCTION

The entry and dispersion of Zika virus (ZIKV) in Brazil has been silent from the beginning of 2014^1^ . However, the severity of the situation surfaced in the second semester of 2015, when an alarming number of cases of microcephaly was registered in the Northeast of the country^2^ . In response to the microcephaly epidemic in Brazil, rapidly associated with ZIKV, the World Health Organization (WHO) declared the ZIKV, at the beginning of 2016, as “a public health emergency of international concern,” highlighting the importance of stronger measures to reduce infection, especially among pregnant women and in fertile age^3^ .

The vertical transmission of the virus was identified as the main cause of the congenital Zika virus syndrome in newborns, since the ZIKV can cross the placental barrier efficiently^4^ , mainly in the first, but also in the second and third trimesters of pregnancy, although with less frequency^5^ .

*Aedes aegypti* was incriminated as the main vector of ZIKV^6^ , a large vector control campaign was instituted throughout the country, called Zika Zero, in order to reduce the infestation levels of this mosquito species^7^ . However, the strategies to control *Ae. aegypti* in Brazil, whether by the mechanical elimination of breeding sites, or by the use of insecticides, have obtained disappointing results, with constant dengue epidemics^8^ and resistance to several classes of insecticides widely distributed^9^ .

Given this scenario, the Ministry of Health (MH) recommended the adoption of personal protective measures against the bites of ZIKV-transmitting mosquitoes, especially for pregnant women. These measures include the use of commercial repellents and complementary mechanical protective measures. The mechanical protection recommended by MH refers to wearing clothing that prevents bites (long-sleeved shirts, coats, socks, pants and long skirts), protective screens on doors and windows and mosquito nets^10^ .

Without vaccine or specific treatment for ZIKV and mainly due to the congenital syndrome associated with ZIKV infection, the focus for transmission control was on preventive measure and health promotion. Based on this assumption, and considering the recommendations of the Ministry of Health disseminated in all media at the time^7 , 11^ , the objective of this study was to assess which personal protection measures were employed by pregnant women residing in the northeastern region of Brazil, the most affected by the epidemic between 2015 and 2016, as well as to identify the factors associated with the non-adoption of preventive measures.

## METHODS

This study was developed in the municipality of Propriá, Sergipe, northeastern region of Brazil (10°13’48”S, 36°50’22’’W), with an estimated population of 28,451 inhabitants (24,390 living in urban and 4,061 in rural areas) and human development index (HDI) of 0.661^12^ . The municipality has 10 basic health units (BHU) responsible for monitoring a defined number of families, with actions to promote, prevent and recover the community health. Two BHUs are located in the rural area and eight are located in the urban area.

The epidemiological situation of the municipality of Propriá was considered of medium risk for dengue transmission, with 1.1% of the properties infested with *Ae. aegypti* larvae in the Index Rapid Survey for *Aedes aegypti* (LIRAa) carried out from 12 to 16 September 2016. Propriá had three reported cases of microcephaly until the end of 2016, with one confirmed case^13^ .

A cross-sectional study was carried out, with a survey of information from pregnant women about the use of personal protective measures against ZIKV infection by *Ae. aegypti* mosquito bites. The interviews were conducted during prenatal care at the BHU of the municipality through a semi-structured form applied by an interviewer between November 2016 and February 2017. Two pilot studies were conducted with women of different age groups to assess the understanding of the questions and estimate the duration of the interview (15 to 30 minutes). After the pilot study, the final form was drawn.

To determine the sample size, the confidence level of 95% and sample error of 5% was considered, while the population parameters p and q were fixed in 50%. The reference for calculating the number of pregnant women was the sum of live births, stillbirths and abortions in 2015 in the database of MH^14^ . A stratified sample was calculated to maintain the proportion of pregnant women living in rural (20%) and urban (80%) areas, resulting in a minimum sample of 174 pregnant women, with 34 living in rural area and 140 in urban areas. The inclusion criteria were pregnant women aged more than 15 years (mean age to finish the elementary school in Brazil), residents in Propriá and who received prenatal care at the BHUs of the Unified Health System (SUS).

### Data Analysis

The collected data were typed (double-typing) in a spreadsheet using Microsoft software^®^ Excel 2013. Data analysis involved descriptive statistics techniques that understood the attainment of absolute and relative frequencies of nominal variables. Bivariate analyses were performed, with crosses between variables using the chi-square or Fisher’s exact test and odds ratio (OR). The confidence level employed was 95%.

A logistic regression model was adjusted, assuming as response variables: no use of repellents, no use of mosquito nets, no use of long clothes, no use of screens, and no use of insecticides. The principal component analysis (PCA) was used to solve multicollinearity problems of the set of 20 independent variables selected. The inclusion criteria were the scores that presented an eigenvalue higher than or equal to 1. The weight (importance) of each variable in the construction of each component was observed according to the coefficients generated for each original variable, and the variables of higher weight were used to name the components. The statistical analyses were conducted on the R programming, version 3.4.0.

### Ethical Aspects of the Study

This study was approved by the Research Ethics Committee of the Federal University of Sergipe (Protocol 1.807.743). All pregnant women were informed of the objectives of the study and invited to sign the informed consent form authorizing their participation and use of the information granted for the purpose of the study. All information was kept confidential to maintain the privacy of the respondent. There was no programmed intervention with the pregnant women, and the norms of the National Health Council of the Ministry of Health established in resolution 466/2012, which regulates research in human beings, were respected.

## RESULTS

Of the total 183 pregnant women approached, five were not included because they were younger than 15 years old (age stipulated to finish elementary school), and one woman refused to participate in the interview. Thus, 177 pregnant women were interviewed, 34 from the rural area and 143 from the urban area. The median age was 25 years (amplitude: 15 to 42), with a predominance of those aged 15 to 25 years (55%). Most women (73%) had less than eight years of schooling (41%) and lived with their partners (70%). Only 10% of the sample had college degree or some college, 28% had paid occupation and most lived in the urban area (80%) ( Table 1 ).

**Table 1:** Demographic and social characteristics and gestational period of the pregnant women interviewed in the municipality of Propriá, state of Sergipe, from November 2016 to February 2017.

Characteristic	Frequency	%
Location		
Rural area	34	20
Urban area	143	80
Color		
White	33	19
Brown	130	73
Black	14	8
Age group (years)		
15–20	51	29
21–25	46	26
26–30	44	25
31–35	22	12
36–40	12	7
41–45	2	1
Marital status		
Married	72	41
Single	52	29
Common-law marriage	51	29
Divorced	2	1
Education level		
Some elementary or middle school	51	29
Elementary school	21	12
Some high school	21	12
High school	66	37
College degree	18	10
Occupational situation		
Employed	49	28
Unemployed	128	62
*Bolsa Família* aid		
Receives benefit	68	38
No benefit	109	62
Gestational age (weeks)		
< 8	25	14
8–12	21	12
12–16	24	13
16–20	27	15
20–24	25	14
24–28	18	10
> 28	37	22

Among the measures recommended by the Ministry of Health, the most used in descending order (n, frequency) were: repellents (100, 57%), mosquito nets (83, 47%), long clothes (78, 44%) and screens on doors and windows (12, 6%). Only 2% of the sample used all the measures recommended by the MH. The alternative measures to the recommended ones were: pyrethroid insecticides (73, 41%), homemade substances used as repellent (38, 21%), mosquito coil (36, 20%), products containing citronella (30, 17%) and plug in repellents (21, 11%). The use of mosquito nets was the most used preventive measure in rural areas (85%), unlike the urban area (37%), while repellents, especially those of active ingredient based on DEET (N, N-diethyl-meta-toluamide) (94%), were more used in the urban environment (59%) than in the rural environment (47%). The use of repellent was proportional to the increase in education level, while the use of mosquito nets was inversely proportional ( Table 2 ).

**Table 2:** Relative frequency of the measures adopted by the pregnant women in the municipality of Propriá, state of Sergipe, according to social criteria.

Variable	Education level						Area			
	
	Elementary School		High school		Higher education		Rural		Urban	
				
	n	%	n	%	n	%	n	%	n	%
Repellents	26	36.0	59	68.0	15	83.0	16	47.0	84	59.0
Mosquito net	42	58.0	37	43.0	4	22.0	29	85.0	54	37.0
Clothing	20	28.0	43	49.0	14	78.0	14	41.0	63	44.0
Screens	0	0.0	10	11.0	2	11.0	3	6.0	9	8.0
Insecticides	21	21.0	42	44.0	10	56.0	10	29.0	33	44.0
Citronella	6	8.0	18	20.0	6	33.0	5	14.0	25	17.0
Homemade products	18	25.0	19	22.0	2	11.0	5	14.0	34	24.0
Plug in repellents	2	3.0	12	14.0	7	39.0	1	1.0	20	14.0
Mosquito coil	17	24.0	16	18.0	3	17.0	3	9.0	33	23.0

Total	72	100.0	87	100.0	18	100.0	34	100.0	143	100.0

The sleeping room was the main place (56%) of the mosquito coil use, and only 8% used in the external areas of the residences. Among the pregnant women who used insecticide pyrethroid spray as a protective medium, 25% used it daily. Among the natural products containing citronella, 59% used incenses, 17% insecticides, 16% candles, 7% topical repellent and 1% cited the cultivation of the plant. Among the homemade substances used as repellent, 24% used alcohol and cloves, 47% cited body moisturizer, 13% ethyl alcohol, 11% body oils and 5% other substances.

The association with change in clothing included: to present record of arbovirosis, have visual contact with a newborn with microcephaly, use of commercial repellent and use of protective screens on doors and/or windows in the residence. Women who altered their routines, avoiding leaving home, had a 3.5 times greater chance of wearing long clothes on their exits. In addition, women with higher incomes (measured by washing machine possession) had six times more chances of having houses with screens ( Table 3 ). Women in the rural area had a higher perception of mosquitoes in their homes and streets [OR = 3.28 (95%CI 1.74–6.18); p > 0.003)] than women in the urban area.

**Table 3:** Relationship between preventive measures and social and behavioral variables.

Variable	Category	Variable		OR (95%CI)	p

		Mosquito net			

		No (%)	Yes (%)		
Formal Education	Elementary school	30 (31.9)	42 (50.6)	0.20 (0.06–0.68)	0.011
	High school	50 (53.2)	37 (44.6)	0.39 (0.12–1.27)	
	Higher education	14 (14.9)	4 (4.8)	1	
Area	Rural	5 (5.3)	29 (34.9)	0.10 (0.04–0.29)	< 0.001
	Urban	89 (94.7)	54 (65.1)	1	

		Long clothes			

Professional guidance	No	64 (64.00)	21 (27.27)	0.21 (0.11–0.40)	< 0.001
	Yes	36 (36.00)	56 (72.73)	1	
Home exits	Yes	14 (14.00)	28 (36.36)	3.51 (1.69–7.29)	0.001
	No	86 (86.00)	49 (63.64)	1	
Commercial repellents	No	54 (54.00)	23 (29.87)	0.36 (0.19–0.68)	0.002
	Yes	46 (46.00)	54 (70.13)	1	
Record of arbovirosis	No	84 (84.00)	54 (70.13)	0.45 (0.22–0.92)	0.042
	Yes	16 (16.00)	23 (29.87)	1	
Baby with microcephaly	No	90 (90.00)	53 (68.83)	0.25 (0.11–0.55)	< 0.001
	Yes	10 (10.00)	24 (31.17)	1	

		Screens			

Washing machine	No	90 (54.55)	2 (16.67)	6.00 (1.28–28.2)	0.014
	Yes	75 (45.45)	10 (83.33)	1	
Commercial repellents	No	76 (46.06)	1 (8.33)	9.39 (1.19–74.4)	0.013
	Yes	89 (53.94)	11 (91.67)	1	
Plug in repellents	Yes	14 (8.48)	7 (58.33)	0.07 (0.02–0.24)	< 0.001
	No	151 (91.52)	5 (41.67)	1	
Long clothes	Yes	68 (41.21)	9 (75)	0.23 (0.06–0.9)	0.032
	No	97 (58.79)	3 (25)	1	
Formal Education	Elementary School	72 (43.64)	0 (0)	-	0.003
	High school	77 (46.67)	10 (83.33)	0.96 (0.19–4.82)	
	Higher education	16 (9.7)	2 (16.67)	1	

		Plug in repellents			

Formal Education	Elementary School	70 (44.87)	2 (9.52)	0.04 (0.01–0.24)	0.002
	High school	75 (48.08)	12 (57.14)	0.25 (0.08–0.78)	
	Higher education	11 (7.05)	7 (33.33)	1	

When the variables were analyzed together through logistic regression analysis, only the first seven components were used as independent variables in the model, maintaining 59.69% of the total variation of the data set, according to Table 4 . The component economic condition of the pregnant woman (PC 1), which included remunerated work, schooling and material goods, indicated that women with low economic power had 2.5 times more chance of not using the repellent, 2.4 times more chance of not using screens in doors or windows, 1.9 times more chance of not wearing long clothes and 1.4 times more chances of not using insecticides than those with higher economic power. Mosquito nets were used as a preventive measure by low-income people. Regarding PC 2 (social condition), the women assisted by the Bolsa Familia Program (BFP – income transfer programme) and with large offspring had 1.7 times more chances of not using repellents compared with primiparous mothers or with few children and not registered in the BFP ( Table 5 ).

**Table 4:** Structure of the independent variables in the composition of the principal components in the regression analysis.

Variable	PC 1	PC 2	PC 3	PC 4	PC 5	PC 6	PC 7
Resides in an urban area	-0.32	0.03	0.13	0.27	**0.38**	-0.14	0.55
Age	-0.16	**0.70**	-0.21	-0.08	0.22	0.23	0.18
Common-law marriage	-0.05	0.13	0.23	0.17	0.01	**0.63**	0.06
Works	**-0.52**	0.16	-0.12	-0.03	0.16	0.44	-0.18
Educational background	**-0.72**	-0.24	-0.08	-0.26	0.11	0.04	-0.05
*Bolsa Família* aid	0.32	**0.75**	0.00	0.17	-0.05	-0.11	-0.02
drinks alcohol	0.07	-0.12	-0.06	**0.75**	0.00	0.02	0.03
Smokes	0.08	0.19	-0.01	**0.74**	0.10	0.02	-0.12
Contact with people with symptoms	0.11	-0.05	-0.03	0.03	**0.68**	0.19	-0.03
Has a health problem	0.01	0.18	0.01	0.03	**0.66**	-0.21	0.10
Has stains on the body	-0.02	-0.27	-0.31	0.19	**0.36**	0.04	-0.33
Saw a baby with microcephaly	-0.02	-0.24	-0.16	-0.16	0.07	**0.59**	0.29
Received professional guidance	-0.04	0.05	**-0.60**	0.07	0.03	0.15	0.31
Fewer home exits due to Zika	0.07	-0.04	-0.27	-0.16	-0.03	0.16	**0.69**
Has a washing machine	**-0.86**	-0.09	-0.02	0.05	-0.14	0.07	0.07
Has a laundry sink	**0.87**	0.08	0.05	0.04	0.12	-0.08	-0.08
Has children	0.15	**0.78**	-0.02	-0.01	0.00	-0.08	-0.09
Satisfactory level of knowledge	-0.25	-0.09	-0.32	-0.02	-0.15	**0.59**	-0.09
Media instruction	-0.09	-0.01	**-0.63**	-0.17	0.22	0.08	-0.11
Month of pregnancy	-0.04	0.13	**-0.65**	0.17	-0.16	-0.13	0.08

PC: principal component

Bold values indicate the principal component in which the variable is inserted.

**Table 5:** Adjustment of the multiple logistic regression model.

Variable	Parameters	OR	Standard error	p
	No use of repellent			

Intercept	-0.590	-	0.212	0.005
PC 1 – Economic condition	0.919	2.506	0.221	0.001
PC 2 – Social condition	0.578	1.782	0.196	0.003
PC 3 – Guidance	0.831	2.296	0.215	0.001
PC 4 – Deleterious habits	0.012	1.012	0.18	0.946
PC 5 – Health condition	0.395	1.484	0.204	0.052
PC 6 – Knowledge about Zika virus	-0.690	0.502	0.233	0.003
PC 7 – Preventive habits	-0.414	0.661	0.200	0.038

	No use of mosquito nets			

Intercept	0.177	-	0.186	0.339
PC 1 – Economic condition	-0.976	0.377	0.203	0.001
PC 2 – Social condition	-0.098	0.907	0.183	0.592
PC 3 – Guidance	0.279	1.322	0.188	0.138
PC 4 – Healthy Habits	0.199	1.220	0.181	0.272
PC 5 – Health condition	0.558	1.747	0.196	0.004
PC 6 – Knowledge about Zika virus	0.15	1.161	0.195	0.442
PC 7 – Preventive habits	0.286	1.331	0.189	0.130

	No change of garment			

Intercept	0.105	-	0.195	0.590
PC 1 – Economic condition	0.666	1.947	0.200	0.001
PC 2 – Social condition	0.124	1.132	0.188	0.510
PC 3 – Guidance	0.912	2.489	0.211	0.000
PC 4 – Healthy Habits	0.542	1.720	0.237	0.022
PC 5 – Health condition	0.079	1.082	0.191	0.679
PC 6 – Knowledge about Zika virus	-0.730	0.482	0.216	0.001
PC 7 – Preventive habits	0.345	0.708	0.193	0.073

	Do not use screens			

Intercept	3.557	-	0.616	0.001
PC 2 – Social condition	0.479	1.614	0.445	0.282
PC 3 – Guidance	0.525	1.691	0.388	0.176
PC 4 – Healthy Habits	1.156	3.173	0.735	0.116
PC 5 – Health condition	0.486	1.626	0.413	0.238
PC 6 – Knowledge about Zika virus	-0.720	0.487	0.291	0.013
PC 7 – Preventive habits	-0.176	0.839	0.324	0.587

	No use of screens			

Intercept	0.400	-	0.169	0.017
PC 1 – Economic condition	0.400	1.492	0.170	0.018
PC 2 – Social condition	-0.147	0.863	0.171	0.389
PC 3 – Guidance	0.127	1.135	0.169	0.452
PC 4 – Healthy Habits	0.115	1.122	0.174	0.507
PC 5 – Health condition	-0.197	0.821	0.168	0.242
PC 6 – Knowledge about Zika virus	0.113	1.119	0.168	0.503
PC 7 – Preventive habits	-0.117	0.837	0.172	0.301

PC 3 (guidance) indicated that women with less than five months of gestation and who did not report having received professional or media education had a 2.2 times higher risk of not using repellent and 2.4 times more chance of not wearing long clothes. PC 4 (deleterious habits) showed that women who consumed alcoholic beverages or smokers had 1.7 times more chances of not modifying the clothing for protection against the *Ae. aegypti* bites *.* PC 5 (health condition) revealed that women with health problems and urban residence had 1.7 times more chances of not using mosquito nets than the group of opposing characteristics.

PC 6 (knowledge about ZIKV) indicated that knowing the disease and having seen infants with microcephaly acted as a stimulator factor for the use of repellents, change of clothing and the use of screens in doors and windows. PC 7 (preventive habits) showed that pregnant women who altered their routines, avoiding leaving home or traveling to relatives’ homes to avoid greater exposure to the vector, also used repellents and worn long clothes ( Table 5 ).

## DISCUSSION

At the end of 2015, the cases of congenital Zika virus syndrome in northeastern Brazil increased, which was rapidly associated with ZIKV infection during pregnancy^3 , 5^ . Worldwide attention has turned to this virus, which has now been seen as a public health problem for pregnant women and their newborn infants^6^ . The ZIKV had a greater impact in the states of Bahia, Pernambuco and Rio Grande do Norte^15^ , where the majority of the population is poor and the climatic conditions are more favorable to the spread of viruses transmitted by mosquitoes^16^ .

However, there is a shortage of epidemiological studies on the use of personal protective measures as prophylaxis for pregnant women against the *Ae. aegypti* bites and consequently against ZIKV infection. In the municipality of Propriá, the professional guidance in prenatal appointments of pregnant women served as motivation and stimulus for the use of repellent, change to longer clothing and information about the disease, while in Florida the awareness and prevention was promoted through billboards that defended the use of barrier protection or mosquito repellants^17^ .

The pregnant women in the rural area reported a higher presence of mosquitoes, due to the greater abundance of vegetation, bodies of water and wasteland or to the greater distance between the houses and consequent higher concentration of mosquitoes. Thus, the use of mosquito nets may have not aimed to avoid the sting of the ZIKV vector, but mosquito bites in general. In addition, it may be related to the low socioeconomic status and lower education level, because mosquito nets are cheaper and more durable measures^18^ . Although *Ae. aegypti* presents daytime habits^8^ , mosquito nets can be a great option to protect young children who spend the most part of the day in the cradle.

While the social and economic components limited the use of repellents by pregnant women in this study, about half of the women with higher income and education level in the state of Texas were concerned about the side effects that these products could cause in their children^19^ (even the DEET, IR3535 and Icaridin were recommended against the infection by ZIKV^20^ ). Although DEET is the most studied insect repellent^21^ , data on pregnancy use are scarce; experiments in laboratory animals did not show congenital problems with their use^22^ .

Pregnant women with higher education level, who received guidance from health professionals, who maintained healthy habits, who met infants with microcephaly and who had some arbovirosis in their life history changed the way they dress, using longer clothing to protect a larger body area against mosquito bites. WHO^23^ recommends light-colored clothing that covers as much of the body as possible (socks, long sleeves, pants or long skirts).

The change in clothing was still related to the reduction in exposure to the external environment; however, women with low social status, without the support of the partner, not only had low adherence to this measure as they were probably still obliged to leave home more often in search of their livelihood. The fact of residing in a city with warm weather also discourages the use of long clothes^24^ .

In the U.S. state of Texas, about a quarter of the women wore shirts or long-sleeved blouses^19^ . In Greece^25^ , country with no report of ZIKV infection, only 16% of pregnant women modified the garment, while the use of repellents reached 53%. In our study, the frequencies of 44% and 57%, respectively, suggested the use of repellents as a complementary measure for wearing long garments, because most of the women who used commercial repellents applied the product only in the arms and legs to prevent stings.

Among all the actions recommended by MH, the use of screens in doors or windows had the lowest adherence among all categories researched, especially women with low economic and social conditions, since this is an onerous measure^18^ .

Most pregnant women who used mosquito coils were unaware of the adverse effects of the present substances. The bedroom was the main place of use, although this type of product should be used in open areas, because it releases smoke, which can cause irritations or respiratory problems indoors^26^ .

In the urban area of Propriá, insecticides were more used than mechanical measures recommended by the Ministry of Health. The practicality of pyrethroid insecticides and the misinformation on toxicity contributed to the low use of screens and mosquito nets in the control of insects in households, although mechanical measures do not cause side effects^17^ and reduce exposure to other unwanted insects. Pyrethroid insecticides can cross the placental barrier and are known to interfere with hormonal and neurological development, in the immune system and in other physiological functions, decreasing the cephalic perimeter of the neonate, for example^27^ . Thus, the insecticides were used to kill insects present in the residences and prevent mosquito bites, without much distinction. Although the role of the indiscriminate household use of insecticides in the selection of resistant populations was generally neglected, its importance was recently indicated as a key factor for the failure to release *Ae. aegypti* infected with *Wolbachia* ^28^ .

Plug in repellents were used less frequently by pregnant women with low socioeconomic status and in rural areas. They were more used among women with higher education, who used them inside the residence in the form of tablets or refill during the night. The home products with repellent function were more used among people with lower education level and income. Commercial repellent prices in Brazil have increased due to the strong demand between 2015 and 2016^29^ , which may have influenced the acquisition of these substances, in addition to misinformation. Alcohol- and clove-based home repellents present low cost and toxicity but have reduced protection time compared with DEET-based topical repellents^30^ .

The recent ZIKV epidemic in the Americas has created a large market with a variety of products for the control and avoidance of mosquitoes^3^ . Among them, there is citronella oil, which showed low toxicity in prenatal development in rodent tests (there are few studies in humans), but has insufficient repelling effect for adequate protection, lasting on average from three to 20 minutes^31^ . Citronella-containing products had no long-lasting repellent effect for any species of mosquito^32^ . Citronella candles and incense, reported by the pregnant women, also did not prove to have sufficient repellent effect^33^ .

The main guideline for the fight against ZIKV in Brazil focused on the vector as responsible for the disease and not the virus^11^ . Thus, all actions were aimed towards the elimination of the mosquito as a personified enemy whose elimination would solve the problem^16^ , with mobilization of the armed forces and task forces of cooperation and education of the population. However, considering that the infestation by *Ae. aegypti* has been strongly related to issues involving basic sanitation, mainly to the supply of drinking water^34^ , vector control strategies disconnected from the confrontation with social challenges may not be the long-term sustainable solution.

The vast majority of cases of congenital Zika syndrome have concentrated in the states of the northeast region^15^ , where access to water and sanitation is limited^34^ . Thus, the concentration of the disease is also related to the irregular and unpredictable supply of water, since the pressure of the pipes decreases depending on how far they are from the central distribution points^16^ . This general situation is aggravated by the region’s characteristic drought, forcing peripheral populations, without the State’s assistance, to store water. Although the ZIKV epidemic has calmed down in 2017, the transmission still persists^35^ and the levels of *Ae. aegypti* infestation remain high. The failures in the implementation of effective collective actions led to the adoption of personal protective measures, and the cost fell especially in the female population.

The images of children affected by microcephaly made women (especially the pregnant ones) the target audience of campaigns^16^ . Women were responsible for adopting preventive measures against Zika, while the call for male participation, if any, was minimized in the process. Thus, the burden of the responsibility fell on the women, especially those with low income, from whom the adoption of personal preventive measures and the procrastination of pregnancy were expected^10^ .

The ZIKV epidemic was a tragedy that largely affected women of lower socioeconomic status^36^ . In a period of deep economic recession in the country^37^ , the financial situation of pregnant women influenced the use of personal protective measures, from the cost of repellents, expenses with clothing, insecticides and other resources to the difficulty of locomotion for the health units, especially in rural areas. However, the measures of collective protection, root of the problem, continue to be neglected^11^ , and the population continue to be blamed for the reduction of vector transmission diseases^16^ . Therefore, the vector control such as *Ae. aegypti* and the use of individual protective measures are only some of the possible strategies to consider when dealing with ZIKV and its relationship with the birth of infants with congenital Zika syndrome. One of the most important lessons taken from this phenomenon is that social iniquity is an underlying factor for the emergence of the disease and perhaps the biggest obstacle to its elimination.
