# Supplementary material for: Personal protective measures of pregnant women against Zika virus infection
Source: Rev Saude Publica. 2019 Aug 23;53:72. doi: 10.11606/s1518-8787.2019053001146 (PMC6707619; doi:10.11606/s1518-8787.2019053001146)
Supplement: Translation

# Translation: Medidas de proteção individual de gestantes contra a infecção pelo zika vírus

**Authors:** Vladimir Antonio Dantas Melo, José Rodrigo Santos Silva, Roseli La Corte

**Affiliations:** I Universidade Federal de Sergipe. Programa de Pós-Graduação em Biologia Parasitária. São Cristovão, SE, Brasil; II Universidade Federal de Sergipe. Departamento de Estatística e Ciências Atuariais. Centro de Ciências Exatas e Tecnologia. São Cristovão, SE, Brasil; III Universidade Federal de Sergipe. Departamento de Morfologia. Centro de Ciências Biológicas e da Saúde. São Cristovão, SE, Brasil

**Keywords:** Gestantes, Infecção pelo Zika virus, prevenção & controle, Infecções por Arbovirus, prevenção & controle, Repelentes de Insetos, Mosquiteiros, utilização, Fatores Socioeconômicos

## Abstract

**OBJETIVO:**

Avaliar a adesão de gestantes às medidas de proteção individual contra picadas de mosquitos, recomendadas pelo Ministério da Saúde e investigar os fatores associados à não adoção dessas medidas.

**MÉTODOS:**

Foram entrevistadas 177 gestantes entre novembro de 2016 e fevereiro de 2017 nas 10 unidades básicas de saúde da cidade de Propriá, SE, duas localizadas na zona rural e oito na zona urbana, durante as consultas de pré-natal, para levantar informações sobre o uso de medidas preventivas contra a transmissão vetorial do zika vírus. Os dados foram analisados utilizando métodos de estatística descritiva, teste do qui-quadrado ou teste exato de Fisher, e foi calculado o *odds ratio* . As variáveis independentes foram agrupadas por meio da análise de componentes principais, e as dependentes (uso de repelentes, mosquiteiros, vestimentas, telas e inseticidas) foram analisadas pelo método de regressão logística.

**RESULTADOS:**

Entre as medidas recomendadas pelo Ministério da Saúde, o uso de mosquiteiros foi a mais utilizada por gestantes residentes na zona rural e de baixa escolaridade, enquanto os repelentes foram mais utilizados por mulheres da zona urbana e com maior tempo de estudo. Mulheres com situação socioeconômica vulnerável apresentaram risco 2,4 vezes maior de não utilizar telas em suas residências, 1,9 vezes maior de não mudar o modo de se vestir e 2,5 vezes maior de não usar repelentes do que gestantes em melhores condições econômicas.

**CONCLUSÕES:**

A condição socioeconômica das gestantes, especialmente entre as mulheres menos favorecidas, influenciou o uso das medidas de proteção contra o zika vírus, desde a compra de repelentes, vestimentas, inseticidas até outros recursos na cidade de Propriá, SE.

## INTRODUÇÃO

Estima-se que a entrada e dispersão do zika vírus (ZIKV) no Brasil tenha se dado, de forma silenciosa, a partir início de 2014^1^ . Porém, a gravidade da situação veio à tona no segundo semestre de 2015, quando um número alarmante de casos de microcefalia foi registrado no Nordeste do país^2^ . Em resposta à epidemia de microcefalia no Brasil, rapidamente associada ao ZIKV, a Organização Mundial da Saúde (OMS) declarou, no início de 2016, o ZIKV como “uma emergência de saúde pública de preocupação internacional”, destacando a importância de medidas mais firmes para reduzir a infecção, especialmente entre as mulheres grávidas e em idade fértil^3^ .

A transmissão vertical do vírus foi apontada como a principal causa da síndrome congênita do zika vírus em recém-nascidos, uma vez que o ZIKV pode atravessar a barreira placentária de forma eficiente^4^ , principalmente no primeiro, mas também no segundo e terceiro trimestres da gestação, embora com menor frequência^5^ .

A via vetorial de transmissão do ZIKV se dá pela picada da fêmea de *Aedes aegypti* infectada^6^ . Por isso, uma grande campanha para o controle do vetor foi instituída em todo país, denominada Zika Zero, com o objetivo de reduzir os níveis de infestação dessa espécie de mosquito^7^ . Porém, as estratégias para controlar *Ae. aegypti* no Brasil, seja pela eliminação mecânica de criadouros, seja pelo uso de inseticidas, têm obtido resultados decepcionantes, com epidemias de dengue constantes^8^ e a resistência a diversas classes de inseticidas amplamente distribuída^9^ .

Diante desse quadro, o Ministério da Saúde (MS) recomendou a adoção de medidas de proteção pessoal contra a picada de mosquitos transmissores do ZIKV, principalmente para a população gestante. Entre essas medidas, estão o uso de repelentes comerciais e medidas de proteção mecânica complementares. A proteção mecânica recomendada pelo MS refere-se ao uso de vestimenta que acarrete menor exposição às picadas (camisas de mangas compridas, casacos, meias, calças e saias longas), de telas de proteção nas portas e janelas e de mosquiteiros^10^ .

Sem vacina ou tratamento específico para o ZIKV e principalmente devido à síndrome congênita associada à infecção pelo ZIKV, o foco para o controle da transmissão esteve na ação preventiva e na promoção da saúde. Partindo deste pressuposto, e considerando as recomendações do Ministério da Saúde difundidas em todos os meios de comunicação à época^7 , 11^ , o objetivo deste trabalho foi avaliar quais medidas de proteção individual foram empregadas por gestantes residentes na região Nordeste do Brasil, a mais atingida pela epidemia entre 2015 e 2016, bem como identificar os fatores associados à não adoção de medidas preventivas.

## MÉTODOS

Esta pesquisa foi desenvolvida no município de Propriá, SE, região Nordeste do Brasil (10°13’48’’S, 36°50’22’’W), com população estimada em 28.451 habitantes (sendo 24.390 da urbana e 4.061 da rural) e índice de desenvolvimento humano (IDH) de 0,661^12^ . A cidade conta com 10 unidades básicas de saúde (UBS) responsáveis pelo acompanhamento de um número definido de famílias, com ações de promoção, prevenção e recuperação da saúde da comunidade. Duas estão localizadas na zona rural e oito na zona urbana.

A situação epidemiológica do município de Propriá foi considerada de médio risco para transmissão de dengue, com 1,1 % dos imóveis infestados com larvas de *Ae. aegypti* no Levantamento de Índice Rápido para *Aedes aegypti* (LIRAa) no período de 12 a 16 de setembro de 2016. Propriá teve três casos notificados de microcefalia até o final do ano de 2016, com um caso confirmado^13^ .

Foi realizado um estudo transversal, com levantamento de informações das gestantes sobre o uso de medidas de proteção pessoal contra a infecção do ZIKV por picadas de mosquitos *Ae. aegypti* . As entrevistas foram conduzidas durante o pré-natal nas UBS do município por meio de formulário semiestruturado aplicado por um entrevistador entre os meses de novembro de 2016 e fevereiro de 2017. Foram realizados dois estudos-piloto com mulheres de diferentes faixas etárias para avaliar o entendimento das questões e estimar a duração da entrevista (15 a 30 minutos). Após o piloto, foi desenhado o formulário final.

Para a determinação do tamanho da amostra, foi considerado o nível de confiança de 95% e erro amostral de 5%, enquanto os parâmetros populacionais p e q foram fixados em 50%. A referência para o cálculo do número de gestantes foi a soma dos nascidos vivos, mortos e abortados do município no ano de 2015 constante na base de dados do MS^14^ . Foi calculada uma amostra estratificada para manter a proporção de gestantes residentes nos meios rural (20%) e urbano (80%), resultando em uma amostra mínima de 174 gestantes, sendo 34 na área rural e 140 na urbana. Os critérios de inclusão foram gestantes com faixa etária superior a 15 anos (idade média para a conclusão do ensino fundamental no Brasil), residentes em Propriá e que realizaram o pré-natal nas UBS do Sistema Único de Saúde (SUS).

### Análise dos Dados

Os dados coletados foram digitados (dupla digitação) em uma planilha eletrônica utilizando o *software* Microsoft^®^Excel 2013. A análise dos dados envolveu técnicas de estatística descritiva que compreenderam a obtenção de frequências absolutas e relativas das variáveis nominais. Foram realizadas análises bivariadas, com cruzamentos entre as variáveis utilizando o teste do qui-quadrado ou exato de Fisher e *odds ratio* (OR). O nível de confiança adotado foi de 95%.

Foi ajustado um modelo de regressão logística, assumindo como variáveis-resposta: não uso de repelentes, não uso de mosquiteiros, não uso de roupas compridas, não uso de telas e não uso de inseticidas. A análise de componentes principais (PC) foi utilizada com o objetivo de resolver problemas de multicolinearidade do conjunto de 20 variáveis independentes selecionadas. Adotaram-se como critério de inclusão os escores que apresentaram autovalor maior ou igual a 1. O peso (importância) de cada variável na construção de cada componente foi observado conforme os coeficientes gerados para cada variável original, sendo as variáveis de maior peso utilizadas para nomear as componentes. As análises estatísticas foram realizadas no programa R, versão 3.4.0.

### Aspectos Éticos da Pesquisa

Este estudo foi aprovado pelo Comitê de Ética em Pesquisa da Universidade Federal de Sergipe (Protocolo 1.807.743). Todas as gestantes foram informadas dos objetivos da pesquisa e convidadas a assinar o termo de consentimento livre esclarecido autorizando a sua participação e utilização das informações concedidas para a finalidade da pesquisa. Todas as informações foram mantidas em sigilo para manter a privacidade da entrevistada. Não houve qualquer tipo de intervenção programada com as gestantes, e foram respeitadas as normas do Conselho Nacional de Saúde do Ministério da Saúde estabelecidas na Resolução 466/2012, que regulamenta pesquisas em seres humanos.

## RESULTADOS

Do total de 183 gestantes abordadas, cinco não foram incluídas por terem idade inferior a 15 anos (idade estipulada pelo término do ensino fundamental) e uma recusou-se a participar da entrevista. Assim, 177 gestantes foram entrevistadas, sendo 34 da zona rural e 143 da zona urbana. A idade mediana foi de 25 anos (amplitude: 15 a 42), com predomínio daquelas com 15 a 25 anos (55%). A maioria (73%) declarou-se parda, apresentava menos de oito anos de estudo (41%) e residia com seus parceiros (70%). Apenas 10% da amostra tinha nível superior completo ou incompleto, 28% tinha ocupação remunerada e a maioria residia na zona urbana (80%) ( Tabela 1 ).

**Tabela 1:** Características demográficas e sociais e período gestacional das gestantes entrevistadas no município de Propriá, SE, de novembro de 2016 a fevereiro de 2017.

Característica	Frequência	%
Local		
Área rural	34	20
Área urbana	143	80
Cor		
Branca	33	19
Parda	130	73
Negra	14	8
Faixa etária (anos)		
15–20	51	29
21–25	46	26
26–30	44	25
31–35	22	12
36–40	12	7
41–45	2	1
Estado civil		
Casada	72	41
Solteira	52	29
União estável	51	29
Divorciada	2	1
Escolaridade		
Ensino fundamental incompleto	51	29
Ensino fundamental completo	21	12
Ensino médio incompleto	21	12
Ensino médio completo	66	37
Ensino superior	18	10
Situação ocupacional		
Empregada	49	28
Desempregada	128	62
Bolsa Família		
Recebe benefício	68	38
Sem benefício	109	62
Idade gestacional (semanas)		
< 8	25	14
8–12	21	12
12–16	24	13
16–20	27	15
20–24	25	14
24–28	18	10
> 28	37	22

Entre as medidas recomendadas pelo Ministério da Saúde, as mais utilizadas em ordem decrescente (n, frequência) foram: repelentes (100, 57%), mosquiteiros (83, 47%), roupas compridas (78, 44%) e telas nas portas e janelas (12, 6%). Apenas 2% da amostra utilizou todas as medidas preconizadas pelo MS. As medidas alternativas às recomendadas foram: inseticidas piretroides (73, 41%), substâncias caseiras utilizadas como repelentes (38, 21%), repelentes em espiral (36, 20%), produtos contendo citronela (30, 17%) e repelentes elétricos (21, 11%). O uso de mosquiteiros foi a medida preventiva mais utilizada na zona rural (85%), diferentemente da urbana (37%), enquanto repelentes, em especial os de princípio ativo à base de DEET (N, N-dimetil-meta-toluamida) (94%), foram mais utilizados no meio urbano (59%) que no meio rural (47%). O uso de repelentes foi proporcional ao aumento da escolaridade, enquanto a utilização de mosquiteiros foi inversamente proporcional ( Tabela 2 ).

**Tabela 2:** Frequência relativa das medidas adotadas pelas gestantes do município de Propriá, SE, de acordo com critérios sociais.

Variável	Escolaridade						Área			
	
	Fundamental		Médio		Superior		Rural		Urbana	
				
	n	%	n	%	n	%	n	%	n	%
Repelentes	26	36,0	59	68,0	15	83,0	16	47,0	84	59,0
Mosquiteiro	42	58,0	37	43,0	4	22,0	29	85,0	54	37,0
Vestuário	20	28,0	43	49,0	14	78,0	14	41,0	63	44,0
Telas	0	0,0	10	11,0	2	11,0	3	6,0	9	8,0
Inseticidas	21	21,0	42	44,0	10	56,0	10	29,0	33	44,0
Citronela	6	8,0	18	20,0	6	33,0	5	14,0	25	17,0
Produtos caseiros	18	25,0	19	22,0	2	11,0	5	14,0	34	24,0
Repelentes elétricos	2	3,0	12	14,0	7	39,0	1	1,0	20	14,0
Repelentes em espiral	17	24,0	16	18,0	3	17,0	3	9,0	33	23,0

Total	72	100,0	87	100,0	18	100,0	34	100,0	143	100,0

O quarto de dormir foi o principal local (56%) de emprego dos repelentes em espiral, e apenas 8% fez uso em áreas externas das residências. Entre as gestantes que utilizaram inseticida piretroide spray como meio de proteção, 25% o fez diariamente. Entre os produtos naturais contendo citronela, 59% fizeram uso dos incensos, 17% de inseticidas, 16% de velas, 7% de repelentes de uso tópico e 1% citaram o cultivo da planta. Entre as substâncias caseiras utilizadas como repelentes, 24% utilizaram álcool e cravo da índia, 47% citaram hidratante corporal, 13% álcool etílico, 11% óleos corporais e 5% outras substâncias.

Estiveram associados à mudança na vestimenta: apresentar histórico de arbovirose, ter contato visual com um recém-nascido com microcefalia, empregar repelentes comerciais e utilizar telas de proteção nas portas e/ou janelas na residência. As mulheres que alteraram suas rotinas, evitando a saída de casa, tiveram chance 3,5 vezes maior de utilizar roupas compridas em suas saídas. Além disso, mulheres com renda mais elevada (medida pela posse de máquina de lavar) apresentaram seis vezes mais chances de ter casas com telas ( Tabela 3 ). Mulheres da zona rural tiveram maior percepção de mosquitos nas suas residências e ruas [OR = 3,28 (IC95% 1,74–6,18); p > 0,003)] que mulheres da zona urbana.

**Tabela 3:** Relação entre medidas preventivas e variáveis sociais e comportamentais.

Variável	Categoria	Variável		OR (IC95%)	p

		Mosquiteiro			

		Não (%)	Sim (%)		
Instrução	1º grau	30 (31,9)	42 (50,6)	0,20 (0,06–0,68)	0,011
	2º grau	50 (53,2)	37 (44,6)	0,39 (0,12–1,27)	
	3º grau	14 (14,9)	4 (4,8)	1	
Área	Rural	5 (5,3)	29 (34,9)	0,10 (0,04–0,29)	< 0,001
	Urbana	89 (94,7)	54 (65,1)	1	

		Roupas Compridas			

Orientação profissional	Não	64 (64,00)	21 (27,27)	0,21 (0,11–0,40)	< 0,001
	Sim	36 (36,00)	56 (72,73)	1	
Saídas de casa	Sim	14 (14,00)	28 (36,36)	3,51 (1,69–7,29)	0,001
	Não	86 (86,00)	49 (63,64)	1	
Repelentes comerciais	Não	54 (54,00)	23 (29,87)	0,36 (0,19–0,68)	0,002
	Sim	46 (46,00)	54 (70,13)	1	
Histórico de arbovirose	Não	84 (84,00)	54 (70,13)	0,45 (0,22–0,92)	0,042
	Sim	16 (16,00)	23 (29,87)	1	
Bebê com microcefalia	Não	90 (90,00)	53 (68,83)	0,25 (0,11–0,55)	< 0,001
	Sim	10 (10,00)	24 (31,17)	1	

		Telas			

Máquina de lavar	Não	90 (54,55)	2 (16,67)	6,00 (1,28–28,2)	0,014
	Sim	75 (45,45)	10 (83,33)	1	
Repelentes comerciais	Não	76 (46,06)	1 (8,33)	9,39 (1,19–74,4)	0,013
	Sim	89 (53,94)	11 (91,67)	1	
Repelentes elétricos	Sim	14 (8,48)	7 (58,33)	0,07 (0,02–0,24)	< 0,001
	Não	151 (91,52)	5 (41,67)	1	
Roupas compridas	Sim	68 (41,21)	9 (75)	0,23 (0,06–0,9)	0,032
	Não	97 (58,79)	3 (25)	1	
Instrução	Fundamental	72 (43,64)	0 (0)	-	0,003
	Médio	77 (46,67)	10 (83,33)	0,96 (0,19–4,82)	
	Superior	16 (9,7)	2 (16,67)	1	

		Repelente elétrico			

Instrução	Fundamental	70 (44,87)	2 (9,52)	0,04 (0,01–0,24)	0,002
	Médio	75 (48,08)	12 (57,14)	0,25 (0,08–0,78)	
	Superior	11 (7,05)	7 (33,33)	1	

Quando as variáveis foram analisadas em conjunto por meio da análise de regressão logística, utilizaram-se apenas os sete primeiros componentes como variáveis independentes no modelo, mantendo 59,69% da variação total do conjunto de dados, de acordo com a Tabela 4 . O componente condição econômica da gestante (PC1), que incluiu trabalho remunerado, escolaridade e bens materiais, indicou que mulheres com baixo poder econômico apresentaram 2,5 vezes mais chance de não usar os repelentes, 2,4 vezes mais de não utilizar telas nas portas ou janelas, 1,9 vezes mais de não utilizar vestimentas compridas e 1,4 vezes mais de não utilizar inseticidas que as de maior poder econômico. Os mosquiteiros foram empregados como medida preventiva pelas pessoas de baixa renda. Com relação ao PC 2 (condição social), as mulheres assistidas pelo Programa Bolsa Família (PBF) e com prole numerosa tinham 1,7 vezes mais chances de não usar repelentes em relação às primigestas ou com poucos filhos e não cadastradas no PBF ( Tabela 5 ).

**Tabela 4:** Estrutura das variáveis independentes na composição dos componentes principais na análise de regressão.

Variável	PC 1	PC 2	PC 3	PC 4	PC 5	PC 6	PC 7
Reside em área urbana	-0,32	0,03	0,13	0,27	**0,38**	-0,14	0,55
Idade	-0,16	**0,70**	-0,21	-0,08	0,22	0,23	0,18
Em união estável	-0,05	0,13	0,23	0,17	0,01	**0,63**	0,06
Trabalha	**-0,52**	0,16	-0,12	-0,03	0,16	0,44	-0,18
Grau de instrução	**-0,72**	-0,24	-0,08	-0,26	0,11	0,04	-0,05
Recebe Bolsa Família	0,32	**0,75**	0,00	0,17	-0,05	-0,11	-0,02
Ingere bebida alcólica	0,07	-0,12	-0,06	**0,75**	0,00	0,02	0,03
Fuma	0,08	0,19	-0,01	**0,74**	0,10	0,02	-0,12
Contato com pessoas com sintomas	0,11	-0,05	-0,03	0,03	**0,68**	0,19	-0,03
Tem problema de saúde	0,01	0,18	0,01	0,03	**0,66**	-0,21	0,10
Tem manchas no corpo	-0,02	-0,27	-0,31	0,19	**0,36**	0,04	-0,33
Viu bebê com microcefalia	-0,02	-0,24	-0,16	-0,16	0,07	**0,59**	0,29
Recebeu orientação profissional	-0,04	0,05	**-0,60**	0,07	0,03	0,15	0,31
Sai menos de casa devido ao zika	0,07	-0,04	-0,27	-0,16	-0,03	0,16	**0,69**
Possui máquina de lavar	**-0,86**	-0,09	-0,02	0,05	-0,14	0,07	0,07
Possui tanque de lavar roupa	**0,87**	0,08	0,05	0,04	0,12	-0,08	-0,08
Tem filho	0,15	**0,78**	-0,02	-0,01	0,00	-0,08	-0,09
Nível de conhecimento satisfatório	-0,25	-0,09	-0,32	-0,02	-0,15	**0,59**	-0,09
Instrução da mídia	-0,09	-0,01	**-0,63**	-0,17	0,22	0,08	-0,11
Mês de gestação	-0,04	0,13	**-0,65**	0,17	-0,16	-0,13	0,08

PC: componente principal

Valores em negrito indicam o componente principal no qual a variável está inserida.

**Tabela 5:** Ajuste do modelo de regressão logística múltiplo.

Variável	Parâmetros	OR	Erro-padrão	p
	Não uso de repelentes			

Intercepto	-0,590	-	0,212	0,005
PC 1 – Condição econômica	0,919	2,506	0,221	0,001
PC 2 – Condição social	0,578	1,782	0,196	0,003
PC 3 – Orientação	0,831	2,296	0,215	0,001
PC 4 – Hábitos deletérios	0,012	1,012	0,18	0,946
PC 5 – Condição de saúde	0,395	1,484	0,204	0,052
PC 6 – Conhecimento sobre o zika vírus	-0,690	0,502	0,233	0,003
PC 7 – Hábitos de prevenção	-0,414	0,661	0,200	0,038

	Não uso de mosquiteiros			

Intercepto	0,177	-	0,186	0,339
PC 1 – Condição econômica	-0,976	0,377	0,203	0,001
PC 2 – Condição social	-0,098	0,907	0,183	0,592
PC 3 – Orientação	0,279	1,322	0,188	0,138
PC 4 – Hábitos saudáveis	0,199	1,220	0,181	0,272
PC 5 – Condição de saúde	0,558	1,747	0,196	0,004
PC 6 – Conhecimento sobre o zika vírus	0,15	1,161	0,195	0,442
PC 7 – Hábitos de prevenção	0,286	1,331	0,189	0,130

	Não mudança da vestimenta			

Intercepto	0,105	-	0,195	0,590
PC 1 – Baixa condição econômica	0,666	1,947	0,200	0,001
PC 2 – Condição social	0,124	1,132	0,188	0,510
PC 3 – Orientação	0,912	2,489	0,211	0,000
PC 4 – Hábitos saudáveis	0,542	1,720	0,237	0,022
PC 5 – Condição de saúde	0,079	1,082	0,191	0,679
PC 6 – Conhecimento sobre o zika vírus	-0,730	0,482	0,216	0,001
PC 7 – Hábitos de prevenção	0,345	0,708	0,193	0,073

	Não uso de telas			

Intercepto	3,557	-	0,616	0,001
PC 2 – Condição social	0,479	1,614	0,445	0,282
PC 3 – Orientação	0,525	1,691	0,388	0,176
PC 4 – Hábitos saudáveis	1,156	3,173	0,735	0,116
PC 5 – Condição de saúde	0,486	1,626	0,413	0,238
PC 6 – Conhecimento sobre o zika vírus	-0,720	0,487	0,291	0,013
PC 7 – Hábitos de prevenção	-0,176	0,839	0,324	0,587

	Não uso de inseticidas			

Intercepto	0,400	-	0,169	0,017
PC 1 – Condição econômica	0,400	1,492	0,170	0,018
PC 2 – Condição social	-0,147	0,863	0,171	0,389
PC 3 – Orientação	0,127	1,135	0,169	0,452
PC 4 – Hábitos saudáveis	0,115	1,122	0,174	0,507
PC 5 – Condição de saúde	-0,197	0,821	0,168	0,242
PC 6 – Conhecimento sobre o zika vírus	0,113	1,119	0,168	0,503
PC 7 – Hábitos de prevenção	-0,117	0,837	0,172	0,301

O PC 3 (orientação) indicou que mulheres com menos de cinco meses de gestação e que não relataram ter recebido instrução profissional ou midiática possuíam um risco 2,2 vezes maior de não usar repelentes e 2,4 vezes de não utilizar roupas compridas. O PC4 (hábitos deletérios) mostrou que mulheres que consumiam bebidas alcoólicas ou tabagistas tinham 1,7 vezes mais chances de não modificar a vestimenta para proteção contra a picada do *Ae. aegypti.* O PC 5 (condição de saúde) revelou que mulheres com problemas de saúde e com residência urbana possuíam 1,7 vezes mais chances de não usar mosquiteiros que o grupo de características opostas.

O PC 6 (conhecimento sobre ZIKV) apontou que conhecer a patologia e ter visto bebês com microcefalia agiram como fator estimulador para o uso de repelentes, mudança de vestuário e o uso de telas nas portas e janelas. Já o PC 7 (hábitos de prevenção) mostrou que gestantes que alteraram suas rotinas, evitando sair de casa ou viajar para casas de parentes para evitar maior exposição do vetor, também utilizaram repelentes e roupas compridas ( Tabela 5 ).

## DISCUSSÃO

No final de 2015, houve um aumento de casos de síndrome congênita do zika vírus no Nordeste brasileiro, rapidamente associada à infecção por ZIKV durante a gestação^3 , 5^ . A atenção mundial voltou-se para esse vírus, que passou a ser visto como um problema de saúde pública para mulheres grávidas e seus bebês recém-nascidos^6^ . O ZIKV apresentou impacto maior nos estados da Bahia, Pernambuco e Rio Grande do Norte^15^ , onde a maioria da população é pobre e as condições climáticas são mais favoráveis à propagação de vírus transmitidos por mosquitos^16^ .

Há, no entanto, escassez de estudos epidemiológicos sobre o uso das medidas de proteção individual como profilaxia para gestantes contra a picada do *Ae. aegypti* e consequentemente contra a infecção pelo ZIKV. No município de Propriá, a orientação profissional nas consultas pré-natais das gestantes serviu de motivação e estímulo para o emprego dos repelentes, mudança para um vestuário mais comprido e informação sobre a enfermidade, enquanto na Flórida a conscientização e prevenção foi promovida por meio de outdoors que defendiam o uso de proteção de barreira ou repelentes de mosquitos^17^ .

As gestantes da área rural relataram maior presença de mosquitos, seja pela maior abundância de vegetação, corpos d’água e terrenos baldios ou pela maior distância entre as casas e consequente maior concentração de mosquitos. Assim, o uso de mosquiteiros talvez não tenha sido direcionado para evitar a picada do vetor do ZIKV e sim de picadas de mosquitos em geral. Além disso, pode haver relação com a baixa condição socioeconômica e menor tempo de escolaridade, pois mosquiteiros são medidas mais baratas e de maior durabilidade^18^ . Apesar de o *Ae. aegypti* apresentar hábitos diurnos^8^ , os mosquiteiros podem ser uma ótima opção para proteger crianças pequenas que passam uma grande parte do dia no berço.

Enquanto os componentes sociais e econômicos limitaram o uso de repelentes por parte das gestantes neste estudo, cerca de metade das mulheres de renda e escolaridade superior no estado do Texas estavam preocupadas com os efeitos colaterais que esses produtos poderiam causar em seus filhos^19^ (mesmo o DEET, o IR3535 e a icaridina tendo sido recomendados contra a infecção pelo ZIKV^20^ ). Apesar de o DEET ser o repelente de insetos mais estudado^21^ , são escassos os dados sobre o uso na gestação; experimentos em animais de laboratório não evidenciaram problemas congênitos com seu uso^22^ .

Gestantes com ensino superior, que receberam orientação de profissionais de saúde, que mantinham hábitos saudáveis, que conheceram bebês com microcefalia e que tiveram alguma arbovirose em seu histórico de vida modificaram o modo de se vestir utilizando peças mais compridas, com intenção de proteger uma maior área corporal contra a picada de mosquitos. A OMS^23^ recomenda vestuário de preferência de cor clara e que cubram a maior parte possível do corpo (meias, mangas compridas, calças ou saias compridas).

A mudança da vestimenta ainda esteve relacionada com a diminuição da exposição ao ambiente externo; entretanto, mulheres com baixa condição social, sem o apoio do companheiro, não só tiveram baixa adesão a essa medida como provavelmente ainda foram obrigadas a sair mais vezes de casa em busca de sua subsistência. O fato de residir em uma cidade de clima quente também desencoraja o uso de roupas compridas^24^ .

No estado americano do Texas, cerca de um quarto das mulheres usava camisas ou blusas com mangas compridas^19^ . Na Grécia^25^ , paí sem relato de infecção pelo ZIKV, apenas 16% das mulheres grávidas modificaram a vestimenta, enquanto a utilização de repelentes alcançou 53%. Em nosso estudo, as frequências de 44% e 57%, respectivamente, sugeriram o emprego dos repelentes como uma medida complementar ao uso de vestimentas compridas, pois a maioria das mulheres que utilizavam repelentes comerciais aplicavam o produto somente nos braços e pernas para prevenção das picadas.

Entre todas as ações recomendadas pelo MS, o uso de telas nas portas ou janelas teve a menor adesão entre todas as categorias pesquisadas, em especial mulheres com baixa condição econômica e social, já que essa medida é onerosa^18^ .

Gestantes que utilizaram repelentes em espiral, em sua maioria, desconheciam os efeitos adversos das substâncias presentes. O quarto de dormir foi o principal local de uso, embora esse tipo de produto deva ser utilizado em áreas abertas, pois libera fumaça, que pode causar irritações ou problemas respiratórios em ambientes fechados^26^ .

Na zona urbana de Propriá, os inseticidas foram mais empregados que medidas mecânicas preconizadas pelo Ministério da Saúde. A praticidade dos inseticidas piretroides e a desinformação sobre toxicidade contribuíram para a baixa utilização de telas e mosquiteiros no controle de insetos nos domicílios, inobstante medidas mecânicas não causarem efeitos colaterais^17^ e reduzirem a exposição a outros insetos indesejados. Inseticidas piretroides podem atravessar a barreira placentária e são conhecidos por interferir no desenvolvimento hormonal e neurológico, no sistema imunológico e em outras funções fisiológicas, causando a diminuição do perímetro cefálico do neonato, por exemplo^27^ . Dessa forma, os inseticidas foram utilizados com objetivo de matar insetos presentes nas residências e prevenir a picada de mosquitos, sem muita distinção. Embora o papel do uso domiciliar indiscriminado de inseticidas na seleção de populações resistentes tenha sido, em geral, negligenciado, sua importância foi recentemente indicada como fator fundamental para o insucesso na liberação de *Ae. aegypti* infectados com *Wolbachia* ^28^ .

Os repelentes elétricos foram menos frequentes em gestantes com baixa condição socioeconômica e na zona rural. Houve maior emprego por mulheres com ensino superior, que os utilizaram dentro da residência na forma de pastilhas ou refil durante a noite. Já os produtos caseiros com função repelente foram mais utilizados entre pessoas com menor nível de instrução e renda. Houve disparada dos preços de repelentes comerciais no Brasil devido à forte demanda entre 2015 e 2016^29^ , o que pode ter influenciado a adoção dessas substâncias, além da desinformação. Repelentes caseiros à base de álcool e cravo apresentam baixo custo e toxicidade, porém têm tempo de proteção reduzido em comparação com os repelentes tópicos à base de DEET^30^ .

A recente epidemia de ZIKV nas Américas criou um grande mercado com variedade de produtos para o controle e evitação de mosquitos^3^ . Entre eles, há o óleo de citronela, que demonstrou baixa toxicidade no desenvolvimento pré-natal em testes com roedores (existem poucos estudos em humanos), porém apresenta efeito de repelência insuficiente para uma proteção adequada, durando em média de três a 20 minutos^31^ . Produtos contendo citronela não tiveram efeito de repelência duradouro para nenhuma espécie de mosquito^32^ . Velas e incensos de citronela, relatadas pelas gestantes, também não provaram ter efeito de repelência suficiente^33^ .

A orientação principal para o combate ao ZIKV no Brasil focou-se no vetor como responsável pela doença e não o vírus^11^ . Assim, todas as ações foram direcionadas para a eliminação do mosquito como um inimigo personaficado cuja eliminação solucionaria o problema^16^ , havendo mobilização das forças armadas e mutirões de cooperação e educação da população. No entanto, considerando que a infestação por *Ae. aegypti* tem se mostrado fortemente relacionada a questões envolvendo saneamento básico, principalmente ao abastecimento de água potável^34^ , estratégias de controle vetorial desconectadas do enfrentamento dos desafios sociais podem não ser a solução sustentável em longo prazo.

A grande maioria dos casos de síndrome congênita do zika vírus se concentrou nos estados da região Nordeste^15^ , onde o acesso à água e ao saneamento estão limitados^34^ . Dessa forma, a concentração da doença está também relacionada ao fornecimento irregular e imprevisível de água, uma vez que a pressão das tubulações diminui quanto mais longe se vive dos pontos centrais de distribuição^16^ . Essa situação geral é agravada pela seca caracteristica da região, obrigando as populações periféricas, sem assistência do Estado, a armazenar água. Embora a epidemia de ZIKV tenha arrefecido em 2017, a transmissão ainda persiste^35^ e os níveis de infestação por *Ae. aegypti* continuam elevados. As falhas na implantação de ações coletivas efetivas levaram à necessidade da adoção de medidas de proteção individual, e o custo recaiu especialmente na população feminina.

As imagens de crianças afetadas por microcefalia fizeram das mulheres (especialmente as grávidas) o público-alvo das campanhas^16^ . Coube às mulheres a responsabilidade pela adoção de medidas preventivas contra a zika, enquanto o apelo à participação masculina, se houve, foi minimizado no processo. Assim, o peso da responsabilidade foi colocado sobre as mulheres, em especial as de baixa renda, de quem foi esperada a adoção de medidas preventivas individuais e a procrastinação da gravidez^10^ .

A epidemia de ZIKV foi uma tragédia que atingiu majoritariamente mulheres de situação socioeconômica mais baixa^36^ . Em um período de profunda recessão econômica do país^37^ , a situação financeira das gestantes influenciou no uso das medidas de proteção individual, desde o custo dos repelentes, gastos com vestimentas, inseticidas e outros recursos até a dificuldade de locomoção para as unidades de saúde, em especial na zona rural. Entretanto, as medidas de proteção coletiva, raiz do problema, continuam sendo negligenciadas^11^ , e a população sendo responsabilizada pela redução das doenças de transmissão vetorial^16^ . Portanto, o controle de vetores como o *Ae. aegypti* e a utilização de medidas de proteção individual são apenas algumas das possíveis estratégias a considerar ao lidar com o ZIKV e sua relação com o nascimento de bebês com síndrome congênita do zika vírus. Uma das mais importantes lições tiradas desse fenômeno é que a iniquidade social é um fator subjacente para o surgimento da doença e talvez o maior obstáculo para sua eliminação.
